# Alpha-1-B glycoprotein (A1BG) inhibits sterol-binding and export by CRISP2

**DOI:** 10.1016/j.jbc.2024.107910

**Published:** 2024-10-19

**Authors:** Ola El Atab, Barkha Gupta, Zhu Han, Jiri Stribny, Oluwatoyin A. Asojo, Roger Schneiter

**Affiliations:** 1 Department of Biology, University of Fribourg, Chemin du Musée 10, Fribourg, Switzerland; 2 Dartmouth Cancer Center, Lebanon, New Hampshire, USA

**Keywords:** CAP protein superfamily, cysteine-rich secretory protein (CRISP), immunoglobulin domain (Ig), *Saccharomyces cerevisiae,* snake venom toxins, oprin, sterol export, protein-protein interactions

## Abstract

Proteins belonging to the CAP superfamily are present in all kingdoms of life and have been implicated in various processes, including sperm maturation and cancer progression. They are mostly secreted glycoproteins and share a unique conserved CAP domain. The precise mode of action of these proteins, however, has remained elusive. *Saccharomyces cerevisiae* expresses three members of this protein family, which bind sterols *in vitro* and promote sterol secretion from cells. This sterol-binding and export function of yeast Pry proteins is conserved in the mammalian cysteine-rich secretory protein (CRISP) proteins and other CAP superfamily members. CRISP3 is an abundant protein of the human seminal plasma and interacts with alpha-1-B glycoprotein (A1BG), a human plasma glycoprotein that is upregulated in different types of cancers. Here, we examined whether the interaction between CRISP proteins and A1BG affects the sterol-binding function of CAP family members. Coexpression of A1BG with CAP proteins abolished their sterol export function in yeast and their interaction inhibits sterol-binding *in vitro*. We map the interaction between A1BG and CRISP2 to the third of five repeated immunoglobulin-like domains within A1BG. Interestingly, the interaction between A1BG and CRISP2 requires magnesium, suggesting that coordination of Mg^2+^ by the highly conserved tetrad residues within the CAP domain is essential for a stable interaction between the two proteins. The observation that A1BG modulates the sterol-binding function of CRISP2 has potential implications for the role of A1BG and related immunoglobulin-like domain containing proteins in cancer progression and the toxicity of reptile venoms containing CRISP proteins.

The CAP domain constitutes the defining feature of a large protein superfamily with members found in all kingdoms of life. This superfamily derives its name from its founding constituents: Cysteine-rich secretory proteins (CRISPs) within the mammalian reproductive tract, antigen 5 (Ag5) within the venom secretory ducts of stinging insects, and pathogenesis-related protein-1 (PR-1), which is induced in plants upon pathogen infection (1, 2, 3). Additionally known as sperm coating proteins or TAPS (Tpx-1/Ag5/PR-1/Sc7), these proteins share a conserved CAP domain characterized by a stable three-layered αβα sandwich fold (4). Central to this domain are tandem histidine residues flanked by acidic amino acid side chains (Glu-His, Glu-His), known as tetrad residues, which coordinate binding of a divalent cation such as Zn^2+^ or Mg^2+^ (5, 6, 7, 8). CAP superfamily members are mostly secreted glycoproteins and have been implicated in many fundamental biological processes including immune defense in mammals and plants, sperm maturation and fertilization, prostate and brain cancer, pathogen virulence, and venom toxicity. Even though these proteins are extensively studied, their precise mode of action remains to be defined (1, 2, 3, 9, 10).

CRISP2 is a notable member of the CRISP family, predominantly associated with male reproductive biology, and plays an essential role in sperm maturation and fertilization (1, 11, 12). Mammalian CRISP proteins typically comprise an N-terminal CAP domain and a C-terminal cysteine-rich domain, also referred to as ion channel regulatory domain, linked by a hinge region (13, 14, 15). Dysregulation of certain CRISP proteins in malignant cells hints at their potential roles in cancer progression; for instance, CRISP3 is significantly upregulated in prostate cancer, where it serves as a diagnostic and prognostic marker, and cholesterol has a likely role in the progression to advanced disease (16, 17). CRISP-like proteins found in reptiles and insect venoms exhibit neurotoxin-like effects and block angiogenesis, highlighting their diverse functionalities (18).

Alpha-1-B glycoprotein (A1BG) is a human plasma glycoprotein of unknown function (19, 20). The protein harbors five repeating structural domains of about 92 to 98 amino acids each, with sequence similarity to the variable region of members of the immunoglobulin (Ig) superfamily (21). A1BG is thus a member of the Ig superfamily, which serves diverse functions based on molecular recognition especially in the immune system and cell adhesion (22, 23). A1BG has been identified as an autoantigen in patients with rheumatoid arthritis and is overexpressed in rheumatoid arthritis, pancreatic ductal adenocarcinoma, bladder, breast and lung cancer, pediatric patients with steroid resistant nephrotic syndrome and liver cancer cell lines, suggesting that A1BG might be a cancer associated gene and a novel tumor marker (24, 25, 26, 27, 28, 29, 30). The glycosylation pattern of A1BG has been suggested to play a crucial role in its functional diversity, influencing interactions with other proteins and cellular compartments (26, 31). Despite its association with various health conditions, the specific molecular mechanisms underlying A1BG's participation in these processes remain incompletely understood.

CRISP3 has been shown to bind A1BG with nanomolar affinity, forming a stable, noncovalent, equimolar stoichiometric complex (32). Complex formation between A1BG and CRISP3 appears to be conserved as serum A1BG across different mammals, including cow, horse, and rabbit, all bind human CRISP3, suggesting potential functional significance (33). The sterol-binding function of CRISP proteins has been implicated in cholesterol metabolism and cancer progression (34, 35). However, the precise role of A1BG, a known interactor of CRISP proteins, in modulating this function remains unexplored. This study aims to characterize the inhibitory effect of A1BG on CRISP2 sterol-binding and export and investigate the molecular basis of this interaction. We demonstrate that coexpression of A1BG with CRISP2 or CRISP3 impedes the sterol export function of CRISP proteins *in vivo* without affecting their secretion. Furthermore, purified A1BG binds CRISP2 with nanomolar affinity and inhibits sterol binding by CRISP2 *in vitro*. Our structural analysis suggests that the third Ig domain of A1BG (Ig3) engages in high-affinity binding with CRISP2, potentially *via* intermolecular antiparallel β-sheet interactions. Notably, the interaction between A1BG and CRISP2 requires magnesium, indicating the significance of magnesium coordination by the conserved tetrad residues within the CAP domain of CRISP2. We discuss the potential implications of this protein interaction in prostate physiology and an innate immunity against reptile venoms.

## Results

### Expression of A1BG blocks sterol export without affecting the secretion of CRISP2

**Figure 1:**
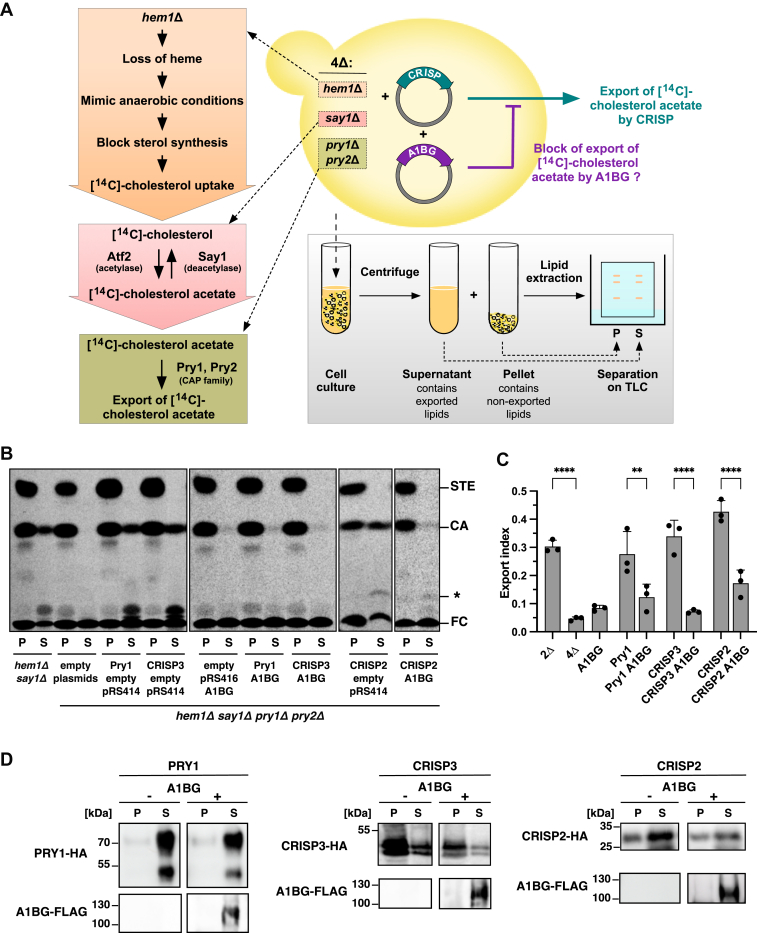
**A1BG inhibits sterol export by CAP family proteins**. *A*, schematic representation of the experimental system. Heme-deficiency of *Saccharomyces cerevisiae* mimics anaerobic conditions and allows for labeling of cells with exogenously supplied [^14^C]-cholesterol. Deletion of the sterol deacetylase Say1 results in the accumulation of acetylated [^14^C]-cholesterol, which is then secreted out of the cells by binding to the yeast CAP proteins Pry1 and Pry2. The block of secretion of [^14^C]-cholesterol acetate of a quadruple mutant (4Δ; *hem1Δ say1Δ pray1Δ pry2Δ*) is complemented by a plasmid-borne copy of a mammalian CAP family proteins such as CRISP. Coexpression of a protein which interacts with CRISP, such as A1BG is being used to assess whether this protein-protein interaction (CRISP-A1BG) affects the sterol binding and export function of CRISP. *B*, export of acetylated cholesterol is blocked in cells expressing A1BG. Acetylation and export of [^14^C]-cholesterol was examined in *hem1Δ say1Δ* double mutant cells and in quadruple mutant cells lacking the endogenous CAP family members Pry1 and Pry2 (*hem1Δ say1Δ pry1Δ pry2Δ*). Strains expressing the indicated CAP proteins (PRY1, CRISP3, or CRISP2) from a plasmid (pRS416) and coexpressing A1BG or bearing an empty control plasmid (pRS414) were cultivated in the presence of [^14^C]-cholesterol. Lipids were extracted from cell pellet (P) and culture supernatant (S), separated by TLC, and visualized by phosphorimaging. The position of free cholesterol (FC), cholesterol acetate (CA), steryl esters (STE), and an unidentified lipid (∗) are indicated to the *right*. *C*, quantification of cholesterol acetate export. The export index represents the relative levels of cholesteryl acetate exported by cells. Export inhibition by A1BG was consistent across three independent experiments, indicating a robust and reproducible interaction between A1BG and CAP family proteins. Data correspond to means ± S.D. of 3 independent experiments and statistical significance is indicated: ∗∗*p* ≤ 0.01; ∗∗∗∗*p* ≤ 0.0001 (one-way ANOVA with Tukey’s *post hoc* test). Precise *p*-values of all statistical analyses are given in Tables S1. *D*, expression of A1BG does not block the synthesis or secretion of CAP family members. Proteins were TCA precipitated from the cell pellet (P) and the culture medium (S) of cells expressing HA-tagged Pry1, CRISP2, or CRISP3 in the presence (+) or absence (−) of FLAG-tagged A1BG and analyzed by Western blotting. Pry1 is detected as a high molecular weight glycosylated protein in the culture supernatant. A1BG fused to the N-terminal signal sequence of pre-pro alpha factor is present as a pre-pro form in the culture supernatant. A1BG, alpha-1-B glycoprotein; CRISP, cysteine-rich secretory protein; TCA, trichloroacetic acid.

To test for sterol binding and export by CAP family proteins, we use an *in vivo* sterol export assay that is based on yeast cells, which are deficient for heme synthesis (34). Heme-deficient *Saccharomyces cerevisiae* mutants (*hem1Δ*) are unable to synthesize ergosterol, the main sterol made by fungal cells, and instead become auxotrophic for sterols (36, 37). This allows for labeling of membrane sterols in *hem1Δ* deficient cells by providing radiolabeled [^14^C]-cholesterol in the culture media. Within the cells, the radiolabeled cholesterol is then subject to an acetylation/deacetylation cycle controlled by the alcohol acetyltransferase 2 and the sterol deacetylase Say1 (38). In the absence of the sterol deacetylase Say1 (*say1Δ*), cholesterol acetate (CA) accumulates and gets eliminated through binding to the yeast CAP family members Pry1 and Pry2, followed by their excretion through the secretory pathway. Thus, in heme-deficient *say1Δ pry1Δ pry2Δ* mutant cells, radiolabeled CA accumulates within the cells rather than in the culture supernatant (34). This block in secretion of acetylated sterols is relieved by expression of mammalian CAP family proteins, such as CRISP2 or CRISP3 (34). This experimental set-up thus allows to test whether proteins that interact with CRISP family members interfere with their sterol binding and export function (35) (Fig. 1*A*).

To examine whether the expression of A1BG would affect the sterol binding and export function of CRISP proteins, a plasmid driving expression of A1BG was transformed into quadruple mutant cells (*4Δ*) lacking Hem1, Say1, Pry1, and Pry2. Cells were then radiolabeled in media containing [^14^C]-cholesterol, washed, and cultured in media containing unlabeled cholesterol to allow for export of acetylated [^14^C]-cholesterol into the culture supernatant. Lipids were then extracted from both the cell pellet and the culture supernatant and separated by TLC (Fig. 1*B*). Radiolabeled lipids were quantified, and the level of acetylated sterols present in the culture supernatant as a fraction of acetylated sterols present in both the cell pellet and the media were plotted as an export index (Fig. 1*C*). These experiments revealed that the coexpression of A1BG with either Pry1, CRISP3, or CRISP2 resulted in a block in sterol secretion by >50% (Fig. 1, *B* and *C*).

To assess whether expression of A1BG would affect the synthesis or secretion of either Pry1, CRISP3, or CRISP2, we coexpressed FLAG-tagged A1BG with hemagglutinin (HA)-tagged Pry1, CRISP3, or CRISP2, then separated yeast cells from the culture medium, precipitated proteins from both fractions with trichloroacetic acid (TCA) and detected the tagged proteins by Western blotting. While HA-tagged Pry1 was detected as a high molecular mass glycoform in the culture supernatant, CRISP2 and CRISP3 were detected in both the cell pellet and the culture supernatant (34). FLAG-tagged A1BG expressed as a fusion with an N-terminal signal peptide derived from pre-pro alpha mating pheromone was present as a high molecular pre-pro form in the culture supernatant, indicating that the protein is indeed secreted (Fig. 1*D*). Our results demonstrate that A1BG inhibits the sterol export function of different CAP family members, including Pry1, CRISP3, and CRISP2, suggesting a conserved regulatory mechanism across species that may play a critical role in cholesterol homeostasis. This effect occurs without impacting CRISP2 synthesis or secretion, highlighting a targeted inhibition of sterol-binding functionality.

### A1BG binds CRISP2 with high affinity and inhibits sterol binding

**Figure 2:**
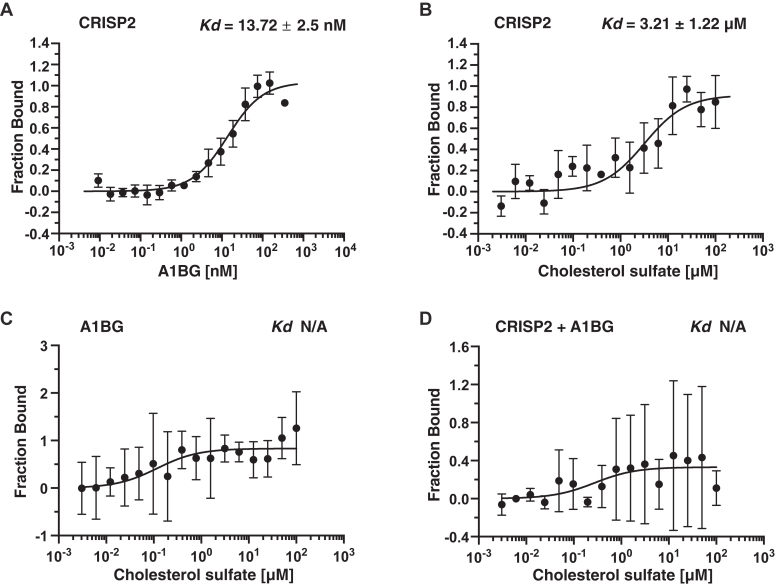
**A1BG binds CRISP2 *in vitro* and blocks sterol binding**. *A*, the direct interaction between A1BG and CRISP2 was assessed by MST. Purified and fluorescently labeled CRISP2 protein was incubated with increasing concentrations of unlabeled A1BG and complex formation was analyzed. MST measurements were performed in triplicates, and the corresponding dissociation constant (*K*_*d*_) is indicated. *B*–*D*, sterol-binding by CRISP2 in the absence (panel *B*) or the presence of A1BG (panel *D*) was assessed by MST. CRISP2 binds cholesterol sulfate in the low micromolar range, but this binding is blocked in the presence of A1BG, which by itself does not bind cholesterol sulfate (panel *C*). Measurements were performed in triplicates and the corresponding dissociation constants (*K*_*d*_) are indicated. N/A; not applicable. A1BG, alpha-1-B glycoprotein; CRISP, cysteine-rich secretory protein; MST, microscale thermophoresis.

To confirm a direct interaction of A1BG with CRISP2, we performed an *in vitro* protein binding assay using microscale thermophoresis (MST). MST was chosen due to its ability to precisely measure binding affinities in real time, even at low protein concentrations, making it ideal for studying interactions like that between A1BG and CRISP2. MST is based on the diffusion of a fluorescently labeled molecule along a microscopic temperature gradient. In this assay, the free protein diffuses faster than its ligand-bound form. Ligands can range from small ions to low molecular weight compounds, liposomes, and even entire viruses (35, 39, 40, 41). Purified and fluorescently labeled CRISP2 was incubated with varying concentrations of A1BG and the interaction between the two proteins was monitored by MST. The results show that A1BG binds CRISP2 with nanomolar affinity (*K*_*d*_ 13.72 ± 2.5 nM) (Fig. 2*A*), which is in accordance with the nanomolar affinity previously determined for the interaction between A1BG and CRISP3 by surface plasmon resonance (*K*_*d*_ 2.1 nM) (32).

To test whether the interaction between A1BG and CRISP2 affects sterol-binding by CRISP2 *in vitro*, we performed lipid binding assays using a water-soluble cholesterol derivative, cholesterol sulfate. While CRISP2 bound cholesterol sulfate in the micromolar range (*K*_*d*_ 3.21 ± 1.22 μM) as reported before (35), A1BG did not (Fig. 2, *B* and *C*). In the presence of an equimolar concentration of A1BG, however, sterol-binding by CRISP2 was blocked (Fig. 2*D*). These results show that complex formation between A1BG and CRISP2 inhibits the propensity of CRISP2 to accommodate sterols, suggesting that, under physiological conditions, A1BG could act as a modulator of lipid binding by CRISP2.

### A1BG does not affect the propensity of CRISP2 to bind and export fatty acids

CAP family proteins have previously been shown to bind eicosanoids and free fatty acids at a second lipid binding site that is independent of their sterol binding site (42, 43, 44). This fatty acid binding site is conserved in CRISP2, and we have previously shown that CRISP2 can export fatty acids *in vivo* (35) (Fig. S1*A*). To test whether A1BG affects fatty acid binding by CRISP2, we measured the affinity of CRISP2 and that of A1BG to palmitic acid by MST. While A1BG did not bind palmitic acid, CRISP2 bound this ligand with micromolar affinity (*K*_*d*_ 64.6 ± 20.7 μM) (Fig. S1, *B* and *C*). This binding affinity of CRISP2 for palmitic acid was not significantly reduced in the presence of A1BG (*K*_*d*_ 70.0 ± 22.7 μM), indicating that A1BG interferes with sterol binding of CAP family proteins, but not with their propensity to bind fatty acids (Fig. S1*D*).

These results were corroborated using an independent *in vivo* fatty acid binding and export assay. This assay is based on the observation that cells lacking the two major acyl-CoA synthases, Faa1 and Faa4, secrete free fatty acids into the medium (45). This lipid export depends on the presence of CAP proteins and is strongly reduced in cells lacking two of the yeast CAP proteins, Pry1 and Pry3 (44, 46). This block in fatty acid export in quadruple mutant cells (*faa1Δ faa4Δ pry1Δ pry3Δ*) is relieved by expression of either Pry1 or CRISP2 and is not impeded by the presence of A1BG, confirming that A1BG does not interfere with the propensity of CRISP2 to bind and export fatty acids (Fig. S1*E*). Taken together, these results suggest that A1BG specifically affects the sterol binding site of the CAP domain but not the fatty acid binding pocket.

### *In silico* dockings predict strong interactions between the Ig3 domain of A1BG and CRISP2

**Figure 3:**
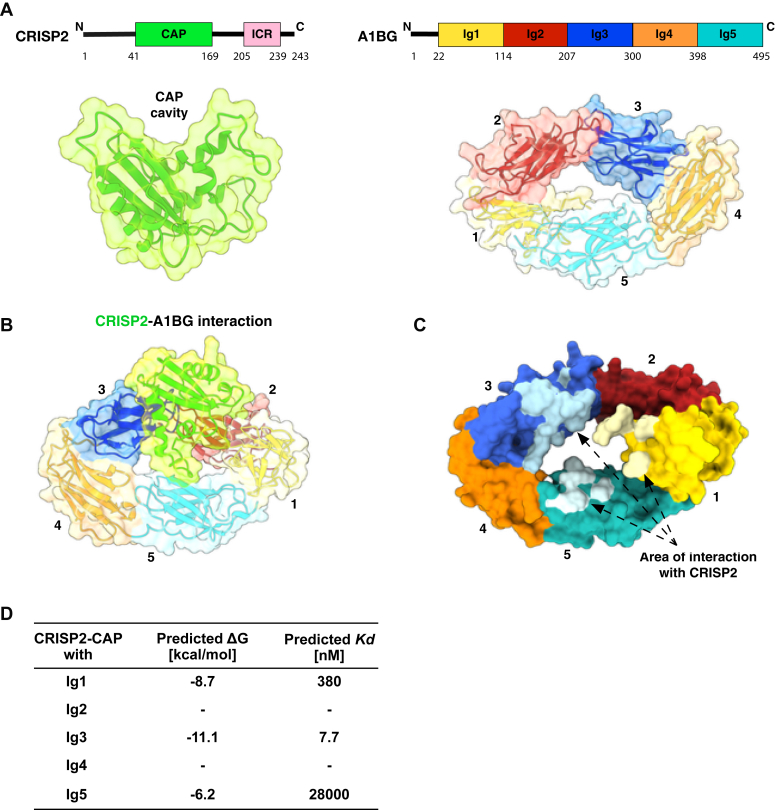
***In silico* docking of CRISP2 to A1BG.***A*, schematic representation of CRISP2 and A1BG. The CAP domain (*green box*) and the ion channel regulatory domain (ICR, *pink box*) of CRISP2 are depicted. The five immunoglobulin (Ig) domains of A1BG are color coded. Amino acid positions of domain boundaries are indicated. The structures of both proteins as predicted by AlphaFold2 are shown below in a combined ribbon/surface representation. The large CAP cavity within the CAP domain of CRISP2 is indicated as is the ring-shaped alignment of the five Ig domains of A1BG. *B*, the predicted interaction of the CAP domain of CRISP2 (*green*) with A1BG is shown using a combined ribbon/surface representation. *C*, the area of surface-to-surface interaction between the CAP domain of CRISP2 and the individual Ig domains of A1BG are indicated in *light* colors. The largest surface interaction occurs between the Ig3 domain of A1BG and the CAP domain of CRISP2, followed by that of Ig1 and Ig5. *D*, table depicting the predicted interaction between CRISP2 and individual Ig domains of A1BG. Free energy (ΔG) and affinities (*K*_*d*_) of interactions between CRISP2 and individual Ig domains of A1BG as predicted by PRODIGY are listed. A1BG, alpha-1-B glycoprotein; CRISP, cysteine-rich secretory protein; PRODIGY, PROtein binDIng enerGY prediction.

To analyze the nature of the molecular interaction between A1BG and CRISP2 in more detail, we performed *in silico* protein docking experiments. Since no experimentally determined structural data for either A1BG or CRISP2 is available, we used AlphaFold2 (https://alphafold.ebi.ac.uk/) to predict the structure of the CAP domain of CRISP2, without its C-terminal channel regulatory domain (47). The predicted structure of the CAP domain of CRISP2 is similar to experimentally determined structures of other CAP family members such as the CAP domain of yeast Pry1 or that of tomato PR1, with RMSD values of 0.773 Å and 0.886 Å, respectively (4, 8). The CAP domain of CRISP2 harbors a conserved surface groove known as the CAP cavity with the tetrad residues implicated in coordinating divalent cations (Fig. 3*A*). In agreement with the sequence homology of A1BG to variable regions of immunoglobulin light and heavy chains, A1BG is predicted to harbor five Ig domains (Ig1-Ig5) and adopt an overall ring-shaped structure (21) (Fig. 3*A*). The Ig domains predicted by AlphaFold2 adopt the characteristic β-sheet fold of Ig superfamily members with a common structural core of 4 β-strands within an antiparallel curled β-sheet sandwich (22). *In silico* dockings between the CAP domain of CRISP2 and A1BG revealed interactions between multiple Ig domains of A1BG and CRISP2 (Fig. 3*B*). Analysis of direct surface-to-surface interactions between these Ig domains and CRISP2 indicate that the Ig3 domain of A1BG displays the largest interaction surface, followed by interactions with Ig1 and Ig5 (Fig. 3, *C* and *D*). Consistent with data obtained from a PRODIGY analysis (PROtein binDIng enerGY prediction), which determines the affinity of protein-protein interactions by predicting its Gibbs free energy (ΔG) and dissociation constant (*K*_*d*_), the interaction between Ig3 and CRISP2 is predicted to result in the lowest ΔG and *K*_*d*_ (ΔG = −11.1 kcal/mol and *K*_*d*_ = 7.7 nM, Fig. 3*D*). These results suggest that the Ig3 domain of A1BG provides the largest contribution to the interaction between the two proteins.

### The third Ig repeat domain of A1BG affects sterol binding by Pry1 and CRISP2 *in vivo*

**Figure 4:**
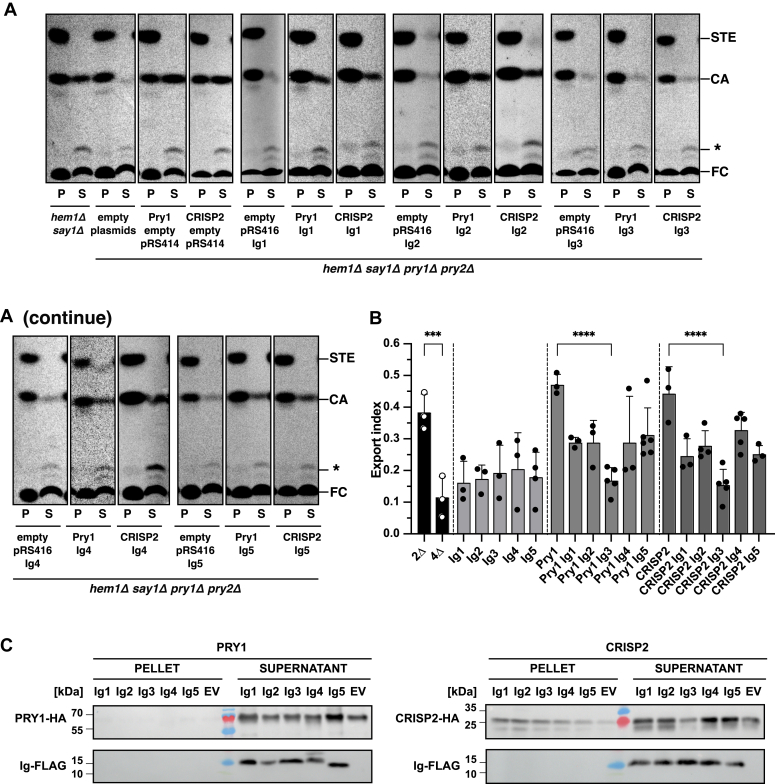
**Expression of the Ig3 domain of A1BG inhibits cholesterol export by Pry1 or CRISP2**. *A*, sterol export by yeast Pry1 and mammalian CRISP2 in the presence of the five different Ig domains, Ig1-Ig5, of A1BG. Quadruple mutant cells (*hem1Δ say1Δ pry1Δ pry2Δ*) expressing different combination of either empty plasmids (pRS414, pRS416), plasmids containing the indicated Ig domains of A1BG, or plasmids for the expression of the CAP proteins Pry1 and CRISP2 were labeled with [^14^C]-cholesterol. Lipids were extracted from both the cell pellet (P) and the culture supernatant (S), separated by TLC, and visualized by phosphorimaging. The position of free cholesterol (FC), cholesterol acetate (CA), steryl esters (STE), and an unidentified lipid (∗) are indicated to the right. *B*, export of radiolabeled cholesterol acetate is plotted as export index in the graph. Data correspond to means ± S.D. of three independent determinations and statistical significance is indicated: ∗∗∗*p* ≤ 0.001; ∗∗∗∗*p* ≤ 0.0001 (one-way ANOVA with Tukey’s *post hoc* test). *C*, expression of the Ig domains of A1BG does not block the synthesis or secretion of the CAP family members Pry1 or CRISP2. Proteins were TCA precipitated from the cell pellet (P) or the culture supernatant (S) of cells expressing HA-tagged Pry1 or CRISP2, in the absence (pRS414, empty plasmid) or the presence of FLAG-tagged Ig domains of A1BG and analyzed by Western blotting. A1BG, alpha-1-B glycoprotein; CRISP, cysteine-rich secretory protein; HA, hemagglutinin; Ig, immunoglobulin; TCA, trichloroacetic acid.

To corroborate these *in silico* docking predictions, we expressed each one of the five Ig domains of A1BG individually in yeast and tested its ability to inhibit the Pry1-and CRISP2-mediated export of cholesterol. This systematic approach revealed that expression of the Ig3 domain of A1BG significantly reduced the export of cholesterol mediated by both tested CAP proteins (Fig. 4, *A* and *B*). The fact that the Ig3 domain of A1BG inhibits cholesterol export of both Pry1 and CRISP2 indicates that the mode of interaction between Ig3 and the CAP domain is conserved. Analysis of the synthesis and secretion of Pry1 and CRISP2 in cells expressing individual Ig domains of A1BG by Western blotting indicated that both CAP family proteins were expressed and secreted, as were the five different Ig domains of A1BG (Fig. 4*C*).

**Figure 5:**
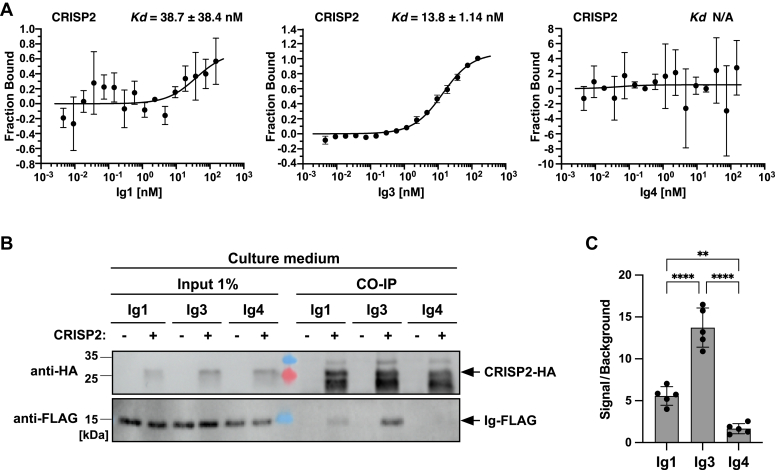
**The Ig3 domain of A1BG displays high affinity interaction with CRISP2 *in vitro* and *in vivo***. *A*, the *in vitro* interaction between three different Ig domains of A1BG, Ig1, Ig3, and Ig4 and CRISP2 was assessed by microscale thermophoresis. Purified and fluorescently labeled CRISP2 protein was incubated with increasing concentrations of unlabeled purified Ig domains of A1BG, Ig1, Ig3, and Ig4, and complex formation was analyzed. Measurements were performed in triplicates and the corresponding dissociation constant (*K*_*d*_) is indicated. N/A; not applicable. *B*, the *in vivo* interaction between three different Ig domains of A1BG, Ig1, Ig3, and Ig4 and CRISP2 was assessed by co-immunoprecipitation. Cells expressing FLAG-tagged Ig domains of A1BG, Ig1, Ig3, or Ig4 (a) and cells coexpressing FLAG-tagged Ig domains together with HA-tagged CRISP2 (b) were cultivated, and the interaction between the Ig domains of A1BG and CRISP2 in the culture medium was analyzed by immunoprecipitation with an anti-HA antibody followed by Western blotting to detect both HA-tagged CRISP2 and FLAG-tagged Ig domains. *C*, quantification of the interaction between the Ig domains of A1BG and CRISP2. The interaction between Ig1, Ig3, or Ig4 and CRISP2 detected by Co-IP were quantified and plotted as ratio between the signal obtained from cells expressing both proteins, CRISP2-HA and Ig-FLAG (+), divided by the background signal from cells lacking CRISP2-HA (−). Data represent mean ± S.D. of 5 determinations and statistical significance is indicated: ∗∗*p* ≤ 0.01; ∗∗∗∗*p* ≤ 0.0001 (one-way ANOVA with Tukey’s *post hoc* test). A1BG, alpha-1-B glycoprotein; CRISP, cysteine-rich secretory protein; HA, hemagglutinin.

To validate these *in vivo* results, we expressed three individual Ig domains of A1BG, Ig1, Ig3, and Ig4 in *Escherichia coli* and purified hexahistidine-tagged versions of these domains. The binding affinities of these Ig domains to CRISP2 were then assessed by MST. Ig1 and Ig3 were chosen as they are both predicted to interact strongly with CRISP2, while Ig4 was used as a negative control that is neither predicted to interact with CRISP2 nor affect its sterol binding function (Figs. 3*D* and 4*B*). Consistent with the predictions from the *in silico* docking analysis, MST measurements revealed a high affinity interaction between Ig3 and CRISP2 (*K*_*d*_ 13.8 ± 1.14 nM) and a weaker interaction with Ig1 (*K*_*d*_ 38.7 ± 38.4 nM), but no detectable interaction with Ig4 (Fig. 5*A*). These interactions between individual Ig domains of A1BG and CRISP2 were confirmed by immunoprecipitation of protein complexes present in the culture supernatant of cells expressing tagged versions of these proteins (Fig. 5*B*). Quantification of these results indicated that immunoprecipitation of the HA-tagged versions of CRISP2 resulted in a significant coprecipitation of FLAG-tagged Ig3, whereas signals of Ig1 and Ig4 remained at much lower levels (Fig. 5*C*). Taken together, these results indicate that the interaction between the Ig3 domain of A1BG and CRISP2 is of high affinity and can be detected both by MST analysis of purified components *in vitro* and in the supernatant of cells expressing tagged versions of these proteins.

### The Ig3 repeat domain of A1BG inhibits cholesterol binding by CRISP2 *in vitro*

**Figure 6:**
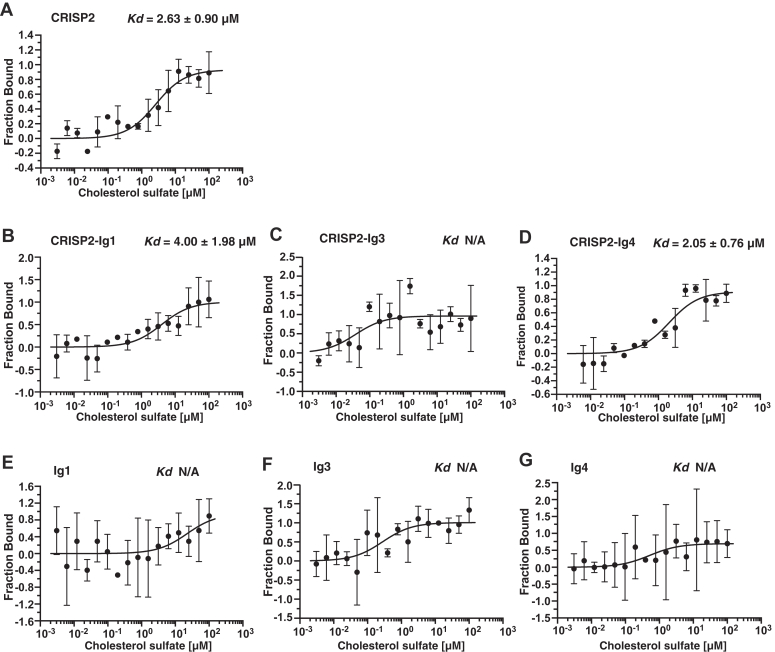
**The Ig3 domain of A1BG blocks sterol binding by CRISP2 *in vitro***. *A*–*D*, sterol-binding by CRISP2 in the absence (panel *A*) or the presence of purified Ig domains of A1BG (panels *B*–*D*) was assessed by microscale thermophoresis. CRISP2 bound cholesterol sulfate in the low micromolar range (panel *A*). Sterol binding by CRISP2 is not affected in the presence of the Ig1 (panel *B*) or Ig4 (panel *D*) domains of A1BG but is blocked in the presence of Ig3 (panel *C*). *E*–*G*, the Ig domains by themselves do not bind cholesterol. Measurements were performed in triplicates and the corresponding dissociation constants (*K*_*d*_) are indicated. N/A; not applicable. A1BG, alpha-1-B glycoprotein; CRISP, cysteine-rich secretory protein; Ig, immunoglobulin.

Given that the Ig3 domain of A1BG is sufficient for high affinity interaction with CRISP2, we next tested whether Ig3 alone could also inhibit the binding of cholesterol by CRISP2 *in vitro* (Fig. 6*A*). MST analysis indicated that the presence of Ig3, but not that of Ig1 or Ig4, was indeed sufficient to block binding of cholesterol by CRISP2 (Fig. 6, *B*–*D*). Control experiments indicated that none of the Ig domains alone could bind cholesterol (Fig. 6, *E*–*G*). These data indicate that binding of the Ig3 domain of A1BG to CRISP2 is sufficient to inhibit the cholesterol binding by CRISP2 *in vitro*.

### A mutant version of CRISP2, CRISP2^L98G Y99P^, affects the interaction with A1BG

**Figure 7:**
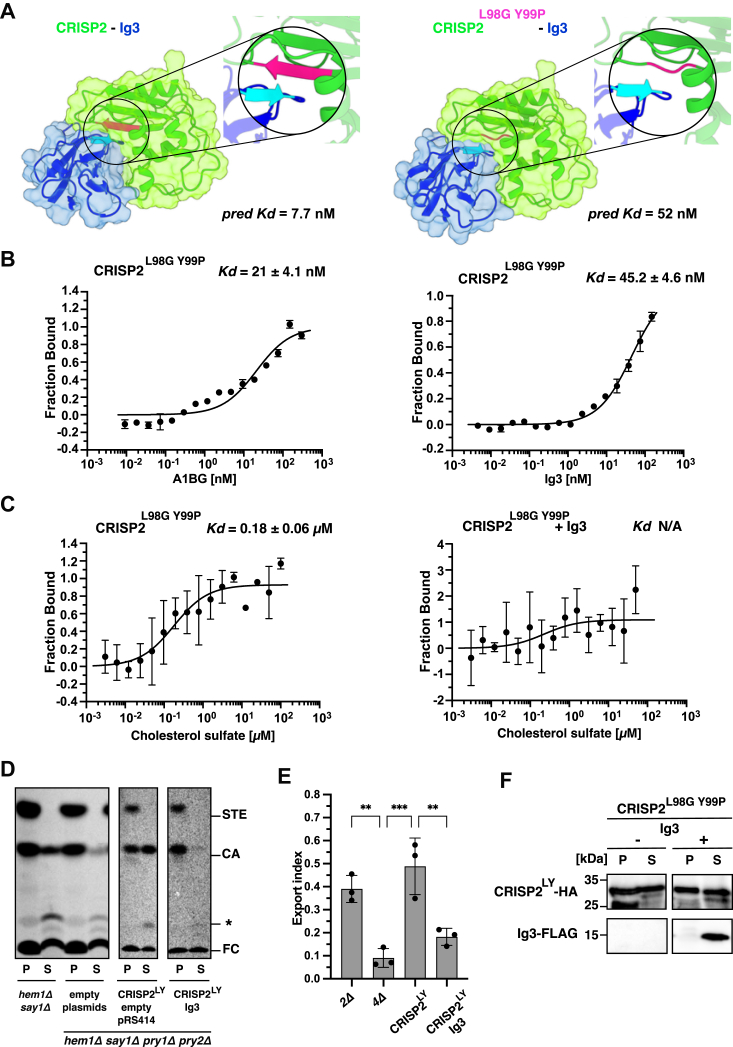
**The interaction between the Ig3 domain of A1BG and CRISP2 is affected by mutations in the β2-strand of CRISP2**. *A*, Model of the interaction between the Ig3 domain of A1BG and CRISP2. The interaction between the Ig3 domain (*blue*) and CRISP2 (*green*) as predicted by *in silico* docking using AlphaFold. The complex between Ig3 and CRISP2 is mainly stabilized by the interaction between a β-sheet of Ig3 (highlighted in *light blue*) and the antiparallel β2-strand of CRISP2 (*pink*) involving the stabilizing residues L98 and Y99. Mutations of both L98 and Y98 to glycine and proline are predicted to disturb the β2-strand of CRISP2 (*right-hand* panel) resulting in a decreased affinity between Ig3 and CRISP2 as indicated by the dissociation constant predicted by PRODIGY (*pred K*_*d*_*)*. *B* and *C*, mutations within the β2-strand residues L98 and Y99 of CRISP2 affect its interaction with A1BG and Ig3. The binding of the CRISP2^L98G Y99P^ double mutant version with A1BG, the Ig3 domain, and cholesterol sulfate was assessed by MST. The presence of Ig3 still inhibited sterol binding by CRISP2^L98G Y99P^. Measurements were performed in triplicates and the corresponding dissociation constants (*K*_*d*_) are indicated. N/A; not applicable. *D*, export of cholesterol by the CRISP2^L98G Y99P^ mutant version is impaired in the presence of Ig3. Quadruple mutant cells (*hem1Δ say1Δ pry1Δ pry2Δ*) expressing CRISP2^L98G Y99P^ and carrying either an empty plasmid (pRS414) or a plasmid containing the Ig3 domains of A1BG were labeled with [^14^C]-cholesterol. Lipids were extracted from both the cell pellet (P) and the culture supernatant (S), separated by TLC, and visualized by phosphorimaging. The position of free cholesterol (FC), cholesterol acetate (CA), steryl esters (STE), and an unidentified lipid (∗) are indicated to the right. *E*, quantification of export of radiolabeled cholesterol acetate is plotted as export index. Data correspond to means ± S.D. of three independent determinations and statistical significance is indicated: ∗∗*p* ≤ 0.01; ∗∗∗*p* ≤ 0.001 (one-way ANOVA with Tukey’s *post hoc* test). *F*, expression of the Ig3 domains of A1BG does not affect the synthesis or secretion of the CRISP2^L98G Y99P^ mutant version in yeast. Proteins were TCA precipitated from either the cell pellet (P) or the culture supernatant (S) of cells expressing HA-tagged CRISP2^L98G Y99P^, in the absence (pRS414, empty plasmid) or the presence of a FLAG-tagged Ig3 domain and analyzed by Western blotting. A1BG, alpha-1-B glycoprotein; CRISP, cysteine-rich secretory protein; Ig, immunoglobulin; HA, hemagglutinin; L98, leucine residue at position 98; MST, microscale thermophoresis; PRODIGY, PROtein binDIng enerGY prediction; TCA, trichloroacetic acid.

To characterize the molecular basis of the binding between CRISP2 and A1BG in more detail, we aimed to identify residues of the two proteins that are important for stabilizing their interaction. A more detailed analysis of the *in silico* predicted interaction between the two proteins indicated that their interaction occurs primarily through an antiparallel β-sheet formed between CRISP2 and A1BG (Fig. 7*A*). A similar type of interaction was previously observed between CRISP2 and prostate secretory protein of 94 amino acids (PSP94), a major protein component in the seminal plasma (35). To test the functional importance of residues within the second β2-strand of CRISP2, we tested whether mutations in two key residues within this structure, namely the leucine residue at position 98 (L98) and the following tyrosine residue (Y99), would affect the interaction between CRISP2 and A1BG. A CRISP2^L98G Y99P^ point mutant version still interacted with the Ig3 domain of A1BG, albeit at lower affinity (*K*_*d*_ 21.0 ± 4.1 nM) than did the WT protein (*K*_*d*_ 13.8 ± 1.14 nM) (Figs 5*A* and 7*B*). As reported before, the CRISP2^L98G Y99P^ point mutant version still bound cholesterol with high affinity (*K*_*d*_ 0.18 ± 0.06 μM), but sterol binding was inhibited in the presence of the Ig3 domain of A1BG (Fig. 7*C*). Additionally, when tested for cholesterol export in the *in vivo* assay, the Ig3 domain of A1BG still inhibited secretion of cholesterol by the CRISP2^L98G Y99P^ mutant version (Fig. 7, *D* and *E*). These results suggest that the mutation of these two interfacial residues does not strongly affect the function of Ig3 to inhibit sterol binding by CRISP2 *in vivo*. The CRISP2^L98G Y99P^ point mutant was synthesized and secreted comparable to the WT CRISP2 protein, suggesting that the mutations do not affect the overall folding or stability of the protein (Fig. 7*F*).

### The interaction between CRISP2 and the Ig3 domain of A1BG is dependent on magnesium ions

**Figure 8:**
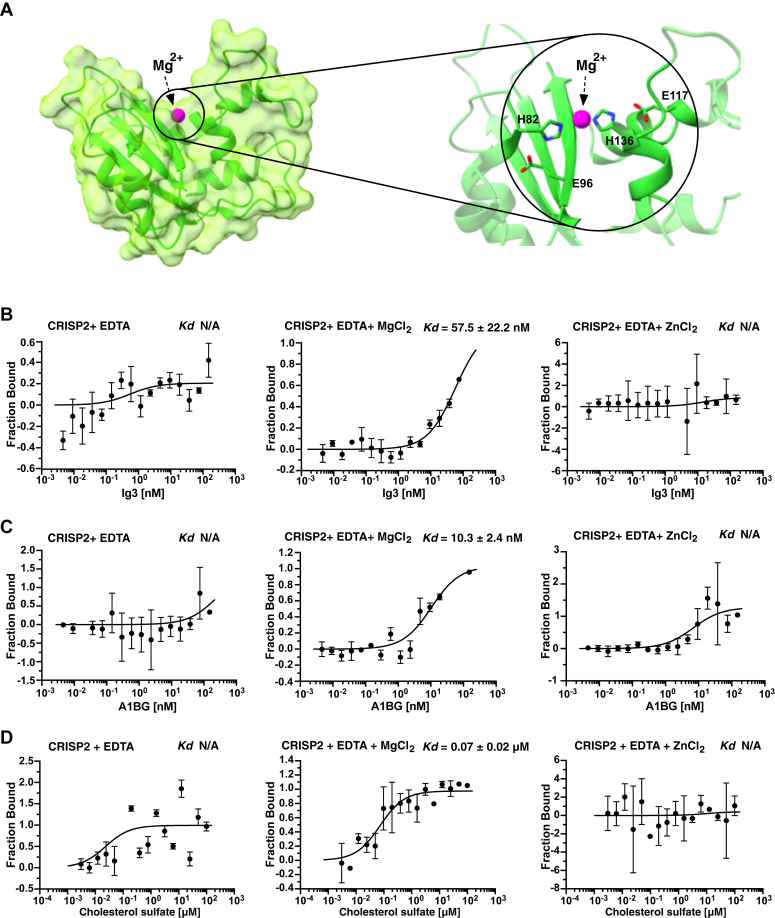
**CRISP2 requires magnesium for binding of Ig3, A1BG, or cholesterol.***A*, model of the CAP domain of CRISP2 showing the highly conserved tetrad residues proposed to coordinate binding of divalent cations. Mg^2+^ is indicated in *magenta*, the conserved tetrad resides (H82, E96, E117, and H136) are indicated. *B* and *C*, binding of the Ig3 (panel *B*) domain of A1BG or that of full-length A1BG (panel *C*) by CRISP2 is dependent on magnesium. Binding of Ig3 or A1BG by CRISP2 was assessed by MST in the presence of EDTA (5 mM), EDTA and MgCl_2_ (10 mM), or EDTA and ZnCl_2_ (20 mM). *D*, binding of cholesterol sulfate by CRISP2 requires magnesium. Binding of cholesterol sulfate by CRISP2 was assessed by MST in the presence of EDTA, MgCl_2_ or ZnCl_2_. All MST measurements were performed in triplicates and the corresponding dissociation constants (*K*_*d*_) are indicated. N/A; not applicable. A1BG, alpha-1-B glycoprotein; CRISP, cysteine-rich secretory protein; Ig, immunoglobulin; MST, microscale thermophoresis.

CAP family proteins have highly conserved tetrad residues with which they can coordinate divalent cations such as Mg^2+^ or Zn^2+^. This cation-coordination is important for the function of these proteins in different physiological settings, including their ability to bind sterols or to inhibit the yeast mating reaction (Fig. 8*A*) (5, 6, 7, 8, 48). To test whether coordination of divalent cations is crucial for the interaction between CRISP2 and the Ig3 domain of A1BG, we tested their interaction in the presence of the magnesium chelator EDTA. Addition of 5 mM EDTA to the MST binding buffer essentially abrogated the interaction between CRISP2 and the Ig3 domain of A1BG (Fig. 8*B*). Protein binding could be restored by the addition of MgCl_2_, thus confirming the cation-dependence of the interaction (Fig. 8*B*). Addition of ZnCl_2_, on the other hand, failed to restored protein binding, indicating that Mg^2+^ is the preferred cation over Zn^2+^ (Fig. 8*B*). This magnesium-dependence of the protein interaction was not only observed with the single Ig3 domain of A1BG but also with full-length A1BG protein (Fig. 8*C*). Lastly, we examined whether cholesterol binding by CRISP2 is dependent on the presence of divalent cations. In the presence of EDTA, CRISP2 failed to bind cholesterol in a dose-dependent manner (Fig. 8*D*). Cholesterol binding could be reconstituted by the back-addition of Mg^2+^ but not by the addition of Zn^2+^, indicating that Mg^2+^ might be the preferred cation for coordination of the highly conserved tetrad residues, which is consistent with the presence of Mg^2+^ in the crystal structure of the CAP domain of yeast Pry1 (Fig. 8*A*) (8). Taken together, these data indicate that CRISP2 interacts with A1BG, particularly through the Ig3 domain of A1BG, and that this interaction depends on the coordination of Mg^2+^ through the highly conserved tetrad residues within the CAP domain of CRISP2.

## Discussion

Here, we focus on the interaction between the mammalian CAP superfamily member CRISP2 and the serum glycoprotein A1BG. A1BG binds CRISP2 and thereby impedes sterol-binding by CRISP2 both *in vivo* and *in vitro.* The nanomolar binding affinity observed between A1BG and CRISP2 (*K*_*d*_ 13.7 nM) indicates a strong and biologically relevant interaction. Such affinities are typically observed in regulatory protein interactions, underscoring the potential physiological importance of this binding in modulating sterol-binding functions. A1BG is composed of five repeated Ig domain, and *in silico* docking predicted strong interactions between A1BG and CRISP2, particularly through the Ig3 domain of A1BG. This finding was validated experimentally, showing that Ig3 alone could inhibit CRISP2-mediated sterol-binding, reinforcing the structural significance of the Ig3 domain in this interaction. Ig3 interacts with CRISP2 through the formation of an intermolecular antiparallel β-sheet, as mutations in conserved amino acids within the β2-strand of CRISP2 decrease binding of A1BG and that of the Ig3 domain of A1BG to CRISP2. We finally show that Mg^2+^-coordination, most likely through the highly conserved tetrad residues within the CAP domain of CRISP2 is required for high affinity interaction with A1BG or the Ig3 domain of A1BG. Interestingly, Zn^2+^ which has also been reported as a divalent cation coordinated by these CAP domain residues does not substitute for the requirement of Mg^2+^ in promoting binding of A1BG or that of cholesterol sulfate to CRISP2 (5, 6, 7, 8).

A1BG is a low abundant serum glycoprotein of unknown function that has been associated with several pathologic conditions (20). A1BG contains five repeated Ig folds, this fold is structurally very stable and shows remarkable plasticity in terms of its binding specificity (21, 22, 23). Proteins that use this fold perform many functions related to immunological recognition and they also associate with several developmental and homeostatic processes (23, 49). Interestingly, the formation of high-affinity heteromeric complexes between small Ig fold-containing secreted proteins appears to be an evolutionarily conserved mechanism to inhibit target protein function. For example, A1BG-like plasma proteins from opossum species form complexes with toxins from reptiles, including snake venom, suggesting a role in neutralizing venom toxicity (50, 51, 52, 53). Oprin, for example, an opossum inhibitor of snake venom metalloproteinases shows high similarity (36% identity) with human A1BG and contains four of the five Ig repeat domains found in A1BG. The presence of oprin in opossum serum may partially account for the resistance of this marsupial to localized effects of rattlesnake envenomation, caused by venom metalloproteinases (50). It is interesting to note that almost all of the known venom toxin inhibitor proteins are associated with the Ig supergene family; for this reason, it has been suggested that these proteins are a derived part of the innate immune system (54, 55, 56). While there are no detectable A1BG homologs in the yeast genome, or reported interactors that might mimic the function of A1BG, we think that the heterologous yeast system can serve as an attractive tool to study the possible physiological consequences of CAP family members and their interactors (3, 10, 57).

Thus, remarkably, two human serum proteins PSP94 and A1BG, both of which show high affinity binding to CRISP have homologs in other species, which function to neutralize the toxicity of CRISP family members from venomous snakes (18, 35, 54, 58). CRISP family proteins from snake venoms exert their neurotoxic function by inhibiting ion channels, through their C terminal cysteine-rich domain (18, 59, 60). Their autotoxicity is efficiently neutralized by binding to an endogenous protein, which belongs to the PSP94 family of proteins (35, 61). Given that complex formation between the Ig repeat containing oprin and related proteins inhibits snake venom toxins and thereby protects the animal from envenomation, complex formation between A1BG and CRISP3 has also been proposed to exert a similar function in mammals by protecting the circulation from a potentially harmful effect of free CRISP3 (32, 62).

Taken together, our results highlight a novel inhibitory mechanism by which A1BG regulates the sterol-binding function of CRISP2. This interaction may be evolutionarily conserved, with broader implications in immune defense and cancer biology (63, 64). Further studies should explore the physiological relevance of this interaction in cancer cells and its potential as a therapeutic target.

## Experimental procedures

### *S. cerevisiae* strains, growth conditions, and plasmids

*S. cerevisiae* double mutant *say1Δ hem1Δ* (*2Δ*) and quadruple mutant *say1Δ hem1Δ pry1Δ pry2Δ* (*4Δ*) strains were generated using PCR deletion cassettes and marker rescue strategies (Table S2). Double mutant strains were grown in yeast peptone dextrose media whereas quadruple mutants were grown in synthetic complete (SC) media. To compensate for the heme deficiency, cells were cultivated either in medium supplemented with delta-aminolevulinic acid (10 μg/ml), or in medium containing cholesterol (20 μg/ml) and Tween-80 (0.05 mg/ml).

Plasmids encoding for CAP proteins were constructed by cloning of PCR amplified fragments from *S. cerevisiae* genomic DNA or from codon optimized synthesized genes (GenScript) into plasmid pRS416, containing an *URA3* selection marker (Table S3). Genes were expressed from an *ADH1* promoter and fused to the signal sequence of pre-pro alpha factor. Codon optimized A1BG and its domains were cloned into pRS414 using hygromycin as selection marker. A1BG full length and its Ig domains were expressed from an *ADH1* promoter and fused either to the signal sequence of Pry1 or that of pre-pro alpha factor.

### *In vivo* sterol export assay

The sterol export assay was performed as described (38). *S. cerevisiae* mutants deficient in heme biosynthesis (*hem1*Δ), lacking the sterol deacetylase enzyme Say1 (*say1*Δ), Pry1 (*pry1Δ*), and Pry2 (*pry2Δ*), were grown overnight in the presence of cold cholesterol/Tween-80. For strains containing pRS414, 80 μg/ml hygromycin was added to the medium. On the second day, cells were harvested by centrifugation, washed twice with SC medium, and then diluted to *A*_600nm_ of 1 into fresh medium containing 0.025 μCi/ml [^14^C]-cholesterol. After overnight growth, cells were washed again with SC medium and cultivated for another day with nonradiolabeled cholesterol containing media. Cells were then centrifuged, and lipids were extracted from both the cell pellet and the culture supernatant using chloroform/methanol (1:1, v/v). Extracted radiolabeled lipids were quantified by scintillation counting, and volumes corresponding to 10,000 cpm (counts per minute) were dried. Dried lipids were resuspended in chloroform/methanol and separated by TLC on silica gel 60 plates (Merck) using the solvent system petroleum ether/diethyl ether/acetic acid (70:30:2, v/v/v). TLC plates were then exposed to phosphorimager screens, and radiolabeled lipids were visualized and quantified using a phosphorimager (GE HealthCare). The sterol export index was calculated as the ratio of extracellular CA to the sum of intracellular and extracellular CA. Export experiments were performed in triplicate, and the export index is given as mean ± SD of three independent experiments. Statistical analysis was performed with one way ANOVA, using Prism (GraphPad Software; https://www.graphpad.com/).

### Fatty acid export

To analyze the CRISP2-dependent export of fatty acids in the presence or absence of A1BG, cells (*faa1Δ faa4Δ pry1Δ pry3Δ*) were grown overnight, the supernatant of 3 *A*_600nm_ units of yeast cells was collected, and lipids were extracted in chloroform:methanol:HCl (50:100:1.5; *v/v*), supplemented with an internal standard (C17:0). The organic phase was collected and dried under a stream of nitrogen. Fatty acid methyl esters (FAMEs) were produced by incubating the dried fractions at 85 °C for 45 min in 1 ml of methanol-sulfuric acid (5% v/v) supplemented with butylated hydroxytoluene (0.01% w/v). The FAMEs were extracted in a mixture containing 1.5 ml of NaCl (0.9% w/v) and 2 ml of hexane, and the upper phase was collected. FAMEs were then resuspended in hexane and separated on an Agilent 7890A gas chromatograph (GC) equipped with a DB-23 capillary column (30 m × 0.25 mm × 0.25 μm) (Agilent Technologies) and quantified relative to an internal standard (C17:0, 10 μg) as described before (44, 46).

### Protein secretion analysis and Western blotting

To analyze the expression and secretion of CAP proteins, they were tagged with a HA tag (YPYDVPDYA) and A1BG or its Ig domains were tagged with a FLAG tag (DYKDDDDK). Epitope-tagged proteins were extracted from 3 *A*_600nm_ units of cells with 0.185 M NaOH (65), followed by precipitation with 10% TCA. To analyze their secretion into the culture supernatant, total proteins from 20 ml culture medium was precipitated with 10% TCA, acetone washed, solubilized in sample buffer, and analyzed by SDS-PAGE and Western blotting. Western blotting was performed using rat anti-HA antibody (rat, 1:2000, Roche #11867423001), and FLAG monoclonal antibody (mouse, 1:2000, Sigma-Aldrich #F3165). As secondary antibodies: goat anti-rat IgG antibody, horseradish peroxidase conjugate (1:10,000, Merck #AP136P) and goat anti-mouse IgG (horseradish peroxidase) conjugates (1:10,000, Bio-Rad #1706516) were used. ECL Prime chemiluminescence substrate (Sigma-Aldrich) was used for signal development and chemiluminescence was detected with an ImageQuant LAS 4000 biomolecular imager (GE HealthCare; https://www.cytivalifesciences.com). Experiments were performed in triplicates with similar results.

### Protein expression and purification

DNA encoding proteins of interest were PCR amplified and cloned into NcoI and XhoI restriction sites of pET22b, which contains a PelB signal sequence to direct the secretion of expressed protein into the periplasmic space. Plasmids were transformed into *E. coli* BL21, and proteins were expressed with a C-terminal polyhistidine-tag. Different induction strategies were used: overnight induction with lactose at 24 °C for CRISP2 and A1BG, and 6 h of induction with 0.4 mM IPTG at 24 °C for the Ig1 and Ig4 domains of A1BG, and at 30 °C for Ig3. Cells were collected, lysed, and the soluble cell lysates were incubated with nickel-nitrilotriacetic acid beads as per the manufacturer instructions (Qiagen). Beads were washed, loaded onto a column, and proteins were eluted with a buffer containing 60 mM NaH_2_PO_4_, 300 mM NaCl, and 300 mM imidazole, pH 8.0. For MST experiments, proteins were applied to Zeba desalting spin columns (Thermo Fisher Scientific), and the buffer was exchanged to 60 mM NaH_2_PO_4_, 300 mM NaCl, pH 8.0. Protein concentration was determined by Lowry assay using the Folin reagent and bovine serum albumin as a protein standard.

### Microscale thermophoresis

To assess protein-protein and protein-lipid interactions, MST experiments were performed using a Monolith NT.115 (Nanotemper Technologies). Proteins were labeled using the RED-tris-NTA His tag protein labeling kit. Subsequently, 100 nM of labeled CRISP2 protein or the L98G Y99P mutant version of CRISP2 were mixed with a serial dilution of unlabeled A1BG, the Ig domains of A1BG, cholesterol sulfate, or palmitic acid, prepared in binding buffer (20 mM Tris pH 7.5, 30 mM NaCl, and 0.05% Triton X-100). Samples were loaded into MST standard capillaries, and MST measurements were performed using an 80% laser power setting. The dissociation constant *K*_*d*_ was obtained by plotting the fraction bound against the logarithm of ligand concentration, using Prism software to fit a binding curve to the data point using the "one site specific" binding option. All MST measurements were performed in triplicate, with each set of experiments including a negative control to rule out nonspecific binding. For binding inhibition and recovery studies, 5 mM EDTA in the presence or absence of 10 mM MgCl_2_ or 20 mM ZnCl_2_ were added to the binding buffer and preincubated with the purified CRISP2 protein before measuring binding with A1BG, the Ig3 domain of A1BG, cholesterol sulfate, or palmitic acid.

### Co-immunoprecipitation analysis

For co-immunoprecipitation analysis, WT CRISP2 was tagged with an HA epitope, and the Ig domains of A1BG, Ig1, Ig3, and Ig4 were fused to a FLAG tag. Yeast strains expressing these proteins were grown overnight in yeast peptone dextrose medium buffered at pH 6.8 (75 mM phosphate/citrate) and collected by centrifugation. The culture medium was concentrated using size-exclusion spin columns (3K MWCO, Pierce, Thermo Fisher Scientific). The concentrated culture supernatant was incubated overnight at 4 °C with anti-HA magnetic beads (Pierce, Thermo Fisher Scientific). Beads were collected, washed with lysis buffer (50 mM Tris–HCl pH 7.5, 100 mM NaCl, 0.1% NP-40, 5% glycerol, protease inhibitor, and 1 mM PMSF), and eluted by the addition of 2× reducing protein sample buffer. Samples were denatured for 10 min at 95 °C and analyzed by SDS-PAGE and Western blotting. Experiments were performed in multiple replicates with similar results.

### Molecular docking

Since the tertiary structure of CRISP2 or A1BG has not yet been experimentally determined, a structure predicted by AlphaFold2 was used (66, 67). Binding of CRISP2 to A1BG was assessed using the AlphaFold tool in UCSF Chimera X (68, 69). The predicted docking sites between the two proteins were analyzed by PROtein binDIng enerGY prediction and visualized using UCSF Chimera X (69, 70).

## Data availability

All data are contained within the manuscript.

## Supporting information

This article contains supporting information (35, 38).

## Conflict of interest

The authors declare that they have no conflict of interest with the content of this article.
