# Supplementary material for: Alpha-1-B glycoprotein (A1BG) inhibits sterol-binding and export by CRISP2
Source: J Biol Chem. 2024 Oct 19;300(12):107910. doi: 10.1016/j.jbc.2024.107910 (PMC11599453; doi:10.1016/j.jbc.2024.107910)
Supplement: Supporting Information [file mmc1.pdf]

Supporting Information

Supporting Tables

Table S1. Complete report of p values from the statistical analyses
Data indicated in the corresponding figures are highlighted in bold.

Figure 1C. Cholesterol acetate export

Tukey's multiple comparisons test	Adjusted P Value
**2Δ vs. 4Δ**	**<0.0001**
2Δ vs. A1BG	0.0002
2Δ vs. Pry1	0.9961
2Δ vs. A1BG Pry1	0.0017
2Δ vs. CRISP3	0.9759
2Δ vs. A1BG CRISP3	<0.0001
2Δ vs. CRISP2	0.0467
2Δ vs. A1BG CRISP2	0.0317
4Δ vs. A1BG	0.9708
4Δ vs. Pry1	0.0001
4Δ vs. A1BG Pry1	0.4537
4Δ vs. CRISP3	<0.0001
4Δ vs. A1BG CRISP3	0.9977
4Δ vs. CRISP2	<0.0001
4Δ vs. A1BG CRISP2	0.0408
A1BG vs. Pry1	0.0009
A1BG vs. A1BG Pry1	0.9653
A1BG vs. CRISP3	<0.0001
A1BG vs. A1BG CRISP3	>0.9999
A1BG vs. CRISP2	<0.0001
A1BG vs. A1BG CRISP2	0.2803
**Pry1 vs. A1BG Pry1**	**0.0085**
Pry1 vs. CRISP3	0.6688
Pry1 vs. A1BG CRISP3	0.0004
Pry1 vs. CRISP2	0.0096
Pry1 vs. A1BG CRISP2	0.1398
A1BG Pry1 vs. CRISP3	0.0002
A1BG Pry1 vs. A1BG CRISP3	0.8593
A1BG Pry1 vs. CRISP2	<0.0001
A1BG Pry1 vs. A1BG CRISP2	0.8749
**CRISP3 vs. A1BG CRISP3**	**<0.0001**
CRISP3 vs. CRISP2	0.2947
CRISP3 vs. A1BG CRISP2	0.0037
A1BG CRISP3 vs. CRISP2	<0.0001
A1BG CRISP3 vs. A1BG CRISP2	0.1581
**CRISP2 vs. A1BG CRISP2**	**<0.0001**

Figure 4B. Impact of Ig domains on cholesterol acetate export

Tukey's multiple comparisons test	Adjusted P Value
**2Δ vs. 4Δ**	**0.0001**
2Δ vs. Pry1	0.9872
2Δ vs. CRISP2	0.9999
4Δ vs. Pry1	<0.0001
4Δ vs. CRISP2	<0.0001
Pry1 vs. CRISP2	>0.9999
Pry1 vs. Pry1 Ig1	0.1729
Pry1 vs. Pry1 Ig2	0.1722
**Pry1 vs. Pry1 Ig3**	**<0.0001**
Pry1 vs. Pry1 Ig4	0.1728
Pry1 vs. Pry1 Ig5	0.1675
CRISP2 vs. CRISP2 Ig1	0.0974
CRISP2 vs. CRISP2 Ig2	0.2210
**CRISP2 vs. CRISP2 Ig3**	**<0.0001**
CRISP2 vs. CRISP2 Ig4	0.7379
CRISP2 vs. CRISP2 Ig5	0.1265

Figure 5C. Ig-CRISP2 interactions detected by CO-IP

Tukey's multiple comparisons test	Adjusted P Value
**Ig1 vs. Ig3**	**<0.0001**
**Ig1 vs. Ig4**	**0.0045**
**Ig3 vs. Ig4**	**<0.0001**

Figure 7E. Cholesterol acetate export of CRISP2-LY mutant

Tukey's multiple comparisons test	Adjusted P Value
**2Δ vs. 4Δ**	**0.0047**
2Δ vs. CRISP2LY	0.4123
2Δ vs. CRISP2LY Ig3	0.0347
**4Δ vs. CRISP2LY**	**0.0007**
4Δ vs. CRISP2LY Ig3	0.4663
**CRISP2LY vs. CRISP2LY Ig3**	**0.0041**

Table S2. *Saccharomyces cerevisiae* strains used in this study

Name	*Relevant Genotype*	Source
RSY6382	*BY4741; MATa his3Δ1 leu2Δ0 met15Δ0 ura3Δ0*	Lab Collection
RSY1853	*MATα his3Δ1 leu2Δ0 lys2Δ0 ura3Δ0 say1::KanMX hem1::LEU2*	Tiwari et al., 2007
RSY6982	*Δfaa1 Δfaa4 Δpry1 Δpry3*	El Atab et al., 2022
RSY6761	*MATa pry1::KanMX say1::HIS pry2::LoxURA3 hem1::LEU2*	Lab collection

Table S3. Plasmids used in this study

Plasmids	Source
pRS416-ADH1 [URA3]	El Atab et al., 2022
pRS416-ADH1-Pry1SS-CRISP3 [URA3]	El Atab et al., 2022
pRS416-ADH1-Pry1SS-CRISP2 [URA3]	El Atab et al., 2022
pRS416-ADH1-prepro alphaSS-Pry1 [URA3]	El Atab et al., 2022
pRS416-ADH1-Pry1SS-CRISP2 L98G Y99P [URA3]	El Atab et al., 2022
pRS416-ADH1-Pry1SS-CRISP3-HA [URA3]	El Atab et al., 2022
pRS416-ADH1-Pry1SS-CRISP2-HA [URA3]	El Atab et al., 2022
pRS416-ADH1-prepro alphaSS-Pry1-HA [URA3]	El Atab et al., 2022
pRS416-ADH1-Pry1SS-CRISP2 L98G Y99P-HA [URA3]	El Atab et al., 2022
pRS414-ADH1 [hph]	El Atab et al., 2022
pRS414-ADH1-Pry1SS-A1BG [hph]	This study
pRS414-ADH1-Pry1SS-Ig1-FLAG [hph]	This study
pRS414-ADH1-prepro alphaSS-Ig2-FLAG [hph]	This study
pRS414-ADH1-prepro alphaSS-Ig3-FLAG [hph]	This study
pRS414-ADH1-prepro alphaSS-Ig4-FLAG [hph]	This study
pRS414-ADH1-Pry1SS-Ig5-FLAG [hph]	This study
pET22b-PelB-Ig1-6XHis [Amp]	This study
pET22b-PelB-CRISP2-6XHis [Amp]	El Atab et al., 2022
pET22b-PelB-AIBG-6XHis [Amp]	This study
pET22b-PelB-Ig3-6XHis [Amp]	This study
pET22b-PelB-Ig4-6XHis [Amp]	This study
pRS425-ADH1-prepro alphaSS-AIBG-FLAG [LEU2]	This study
pET22b-PelB-CRISP2 L98G Y99P-6XHis [Amp]	El Atab et al., 2022
pET22b-PelB-CRISP2 N97P L98G Y99P-6XHis [Amp]	El Atab et al., 2022

Supporting References

Tiwari, R., Koffel, R. and Schneiter, R. (2007) An acetylation/deacetylation cycle controls the export of sterols and steroids from S. cerevisiae. *EMBO J* **26**, 5109-5119

El Atab, O., Kocabey, A. E., Asojo, O. A. and Schneiter, R. (2022) Prostate secretory protein 94 inhibits sterol binding and export by the mammalian CAP protein CRISP2 in a calcium-sensitive manner. *J Biol Chem* **298**, 101600

Supporting Figures

Figure S1. A1BG does not inhibit fatty acid binding of CRISP2 *in vitro* and *in vivo*
(A) Structural alignment of the fatty acid binding pocket of Pry1 (grey) with that of CRISP2 (green). The fatty acid binding pocket is located at the interface between the first and second alpha helix of the CAP domain. Hydrophobic residues that are stabilizing the binding of the fatty acids (in pink) are depicted in the magnification shown on the right-hand side, highlighting that the hydrophobic character of these residues is conserved. In particular, valine at position 227 in Pry1 is substituted by isoleucine at position 111 in CRISP2, and valine at position 254 of Pry1 is substituted by leucine 140 in CRISP2. A multiple sequence alignment showing the conservation (red boxes) of the hydrophobic character of these amino acids in Pry1, 2, CRISP2, 3 and tablysin-15, is shown below.
(B-D) Binding of fatty acids *in vitro* by CRISP2 is not inhibited by the presence of A1BG. Purified and fluorescently labeled CRISP2 (panel B) or A1BG (panel C) were incubated with increasing concentrations of palmitic acid and complex formation was analyzed by MST. Note that A1BG does not bind fatty acids (panel C). Binding of fatty acids by CRISP2 in the presence of A1BG (panel D) was also analyzed by MST. Measurements were performed in triplicates and the corresponding dissociation constants (K_d) are indicated. N/A; not applicable.
(E) A1BG does not inhibit fatty acid binding of CRISP2 *in vivo.* Quadruple mutant cells lacking the acyl-CoA synthetases *FAA1* and *FAA4* and the yeast CAP family members *PRY1* and *PRY3* (*faa1*Δ *faa4*Δ *pry1*Δ *pry3*Δ) expressing different combinations of plasmid-borne copies of *PRY1*, *CRISP2,* and or *A1BG,* or their respective empty vectors (pRS425 or pRS426) were cultivated overnight. The export of fatty acids into the culture medium was then quantified. Data correspond to means ± S.D. of at least 3 independent experiments and statistical significance is indicated: ***, $p \leq 0.001$ and ****, $p \leq 0.0001$. ns; not significant.

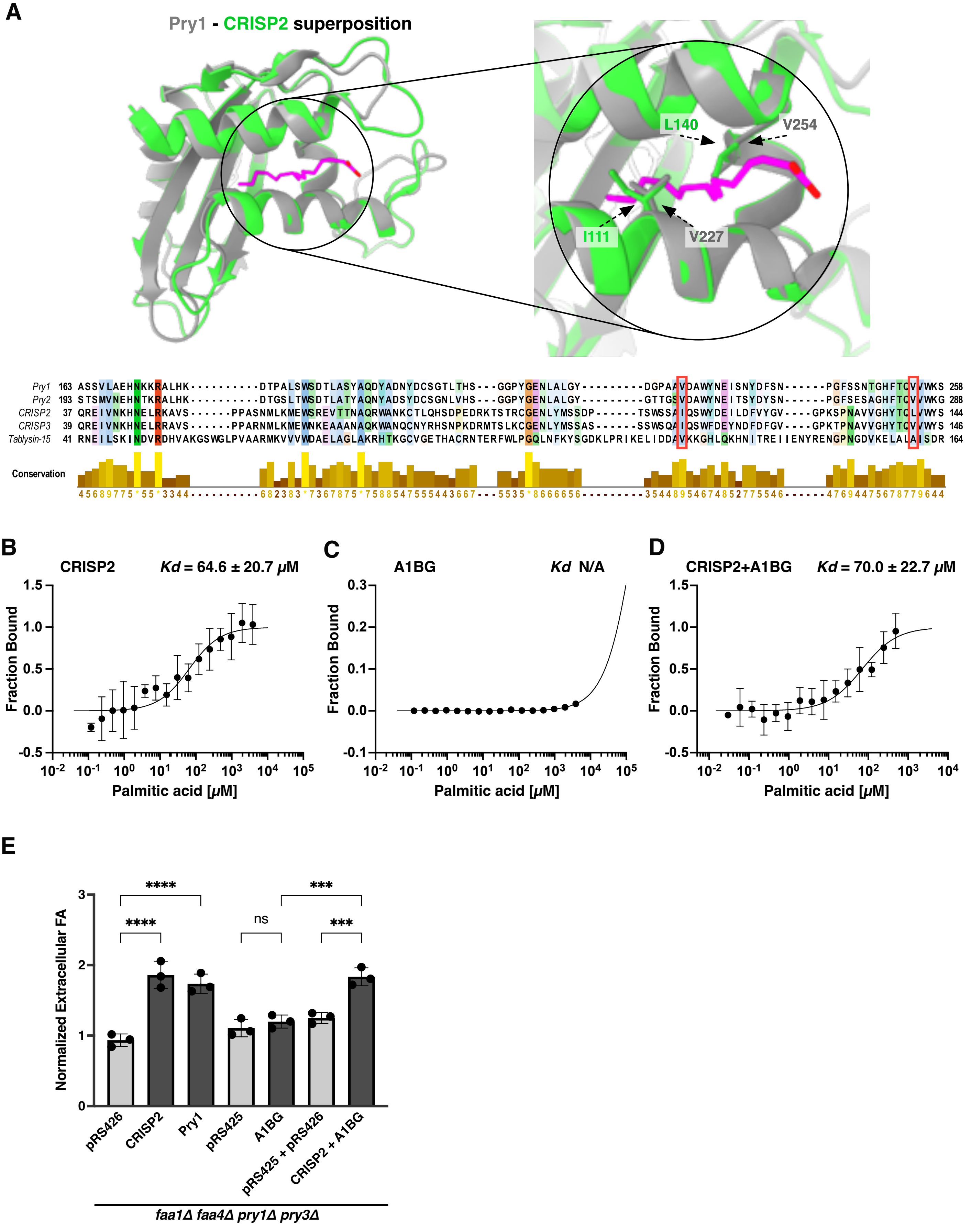
A
Pry1 - CRISP2 superposition
L140
V254
I111
V227
Pry1 163 ASSVLAEHNKKRALHK-----------DTPALSWSDTLASYAQDYADNYDCSGTLTHS---GGPYGENLALGY---------DGPAAVDAWYNEISNYDFSN------PGFSSNTGHFTQVVWKS 258
Pry2 193 STSMVNEHNTKRALHK-----------DTGSLTWSDTLATYAQNYADSYDCSGNLVHS---GGPYGENLALGY---------GTTGSVDAWYNEITSYDYSN------PGFSESAGHFTQVVWKG 288
CRISP2 37 QREIVNKHNELRKAVS------PPASNMLKMEWSREVTTNAQRWANKCTLQHSDPEDRKTSTRCGENLYMSSDP------TSWSSAIQSWYDEILDFVYGV----GPKSPNAVVGHYTQLVWYS 144
CRISP3 39 QREIVNKHNELRRAVS------PPARNMLKMEWNKEAAANAQKWANQCNYRHSNPKDRMTSLKCGENLYMSSAS------SSWSQAIQSWFDEYNDFDFGV----GPKTPNAVVGHYTQVVWYS 146
Tablysin-15 41 RNEILSKINDVRDHVAKGSWGLPVAARMKVVVWDAELAGLAKRHTKGCVGETHACRNTERFWLPGQLNFKYSGDKLPRIKELIDDAVKKGHLQKHNITREIIENYRENGPNGDVKELALAISDR 164
Conservation
45689775*55*3344-----------682383*7367875*7588547555443667---5535*8666656---------35448954674852775546------47694475678779644
B
CRISP2
Kd = 64.6 ± 20.7 μM
Fraction Bound
Palmitic acid [μM]
C
A1BG
Kd N/A
Fraction Bound
Palmitic acid [μM]
D
CRISP2+A1BG
Kd = 70.0 ± 22.7 μM
Fraction Bound
Palmitic acid [μM]
E
Normalized Extracellular FA
****
****
ns
***
***
pRS426
CRISP2
Pry1
pRS425
A1BG
pRS425 + pRS426
CRISP2 + A1BG
faa1Δ faa4Δ pry1Δ pry3Δ

Figure S1
